# Differential pulmonary and circulatory effects of preventive lung protective ventilation in an experimental postoperative sepsis model

**DOI:** 10.1186/cc10716

**Published:** 2012-03-20

**Authors:** J Sperber, M Lipcsey, A Larsson, A Larsson, J Sjölin, M Castegren

**Affiliations:** 1 Centre for Clinical Research Sörmland, Eskilstuna, Sweden; 2 Uppsala University, Uppsala, Sweden

## Introduction

It has been proposed that low tidal volume (VT) ventilation combined with higher PEEP should be used in patients with risk of developing postoperative lung injury instead of the commonly used VT of 10 ml/kg with lower PEEP [1]. Such a ventilatory mode would in theory reduce postoperative lung and organ dysfunction. However, this hypothesis has neither been tested clinically nor experimentally. Therefore we developed an experimental endotoxemic postoperative sepsis model to evaluate the effect of different modes of ventilation.

## Methods

Twenty-five healthy pigs were randomized to three ventilation groups: I: PEEP 10 cmH_2_O, VT 6 ml/kg; II: PEEP 5 cmH_2_O, VT 10 ml/kg, changed to PEEP 10 cmH_2_O, VT 6 ml/kg at the end of laparotomy; III: PEEP 5 cmH_2_O, VT 10 ml/kg. For all groups the plateau pressure was kept below 28 cmH_2_O, normocapnia was reached by respiratory rate and FiO_2 _was adjusted to reach PaO_2 _>12 kPa. Laparotomy for 2 hours was performed to simulate a surgical procedure and then a continuous endotoxin infusion was started at 0.25 μg/kg/hour for 5 hours. Differences between groups were analyzed with ANOVA for repeated measures.

## Results

The groups were equal before and at the end of laparotomy. During the endotoxin infusion, PaO_2_/FiO_2 _was higher in groups I and II than in group III, whereas in pulmonary compliance or functional residual capacity no differences were found. In contrast, group I showed greater negative changes than group III in the circulatory variables; that is, arterial blood pressure, cardiac index, oxygen delivery and oxygen consumption. In all measured variables, group II showed an intermediate response to groups I and III, but no significant differences were found between groups I and II. Groups I and II had slightly higher mean airway pressure at the end of the experiment than group III. However, this does not explain the circulatory differences since they occurred early in the course, temporally different from the continuous slow increase of the airway pressures (*P *≤0.01 ANOVA group by time interaction).

## Conclusion

Low VT ventilation combined with higher PEEP in healthy animals exposed to laparotomy and subsequent experimental post-operative sepsis leads to a less prominent pulmonary dysfunction but to a more hypodynamic circulatory state compared to animals ventilated with a medium-high VT and lower PEEP.
